# Integrated molecular and clinical analysis of BRAF-mutant glioma in adults

**DOI:** 10.1038/s41698-023-00359-y

**Published:** 2023-02-28

**Authors:** Karisa C. Schreck, Pinky Langat, Varun M. Bhave, Taibo Li, Eleanor Woodward, Christine A. Pratilas, Charles G. Eberhart, Wenya Linda Bi

**Affiliations:** 1 Department of Neurology, Johns Hopkins University School of Medicine, Baltimore, MD USA; 2 Department of Oncology, Johns Hopkins University School of Medicine, Baltimore, MD USA; 3 Department of Neurosurgery, Brigham and Women’s Hospital, Harvard Medical School, Boston, MA USA; 4 Department of Pediatrics, Johns Hopkins University School of Medicine, Baltimore, MD USA; 5 Department of Pathology, Johns Hopkins University School of Medicine, Baltimore, MD USA; 6 Dana-Farber Cancer Institute, Harvard Medical School, Boston, MA USA

**Keywords:** CNS cancer, Cancer genetics, Oncogenes

## Abstract

*BRAF* mutations are a significant driver of disease in pediatric low-grade glioma, but the implications of *BRAF* alterations on the clinical course and treatment response in adult glioma remain unclear. Here, we characterize a multi-institutional cohort of more than 300 patients (>200 adults) with *BRAF*-mutated glioma using clinical, pathological/molecular, and outcome data. We observed that adult and pediatric *BRAF*-mutant gliomas harbor distinct clinical and molecular features, with a higher prevalence of BRAF^V600E^ (Class I) and *BRAF* fusions in pediatric tumors. BRAF^V600E^ alterations were associated with improved survival in adults with glioma overall, though not in glioblastoma. Other genomic alterations observed within functional classes were consistent with the putative roles of those *BRAF* mutation classes in glioma pathogenesis. In our adult cohort, *BRAF*^V600E^ alterations conferred sensitivity to targeted therapies. Overall, this large cohort of *BRAF*-altered adult gliomas demonstrates a broad range of molecular alterations with implications for treatment sensitivity and survival.

## Introduction

*BRAF* (v-raf murine viral oncogene homolog B1), a member of the RAF family of serine/threonine protein kinases that signal to MEK-ERK kinases, has been identified as an oncogene driver in multiple cancers. *BRAF* mutations have been classified into three categories by their functional impact on *BRAF* kinase activity, dimerization, and RAS dependency^1,2^. The most common *BRAF* alteration (Class I), p.V600E, functions as a monomer to promote ERK signaling. Less common Class II alterations facilitate ERK signaling through increased homo- and hetero-dimerization of BRAF and do not require upstream activation by RAS or receptor tyrosine kinases (RTKs)^3^. Class III *BRAF* alterations are functionally inactivating alterations that increase the proclivity of BRAF to dimerize with CRAF, thereby increasing downstream ERK signaling^4^. Class I alterations are targetable with FDA-approved small molecule inhibitors, while novel drugs against other *BRAF* alterations and pathway components are under development^5^.

The role of *BRAF* alterations has been a topic of focused interest in pediatric glioma given the high prevalence of p.V600E (Class I) and *BRAF*-*KIAA1549* fusions (Class II)^6,7^, with early evidence of success by *BRAF*-targeted therapy in this group^8–10^. *BRAF* alterations are also observed in adults with low- (5%) and high-grade glioma (3%)^5^, prompting considerable interest in targeting oncogenic *BRAF* alterations in adults^11,12^. To date, our understanding of the spectrum of *BRAF* alterations and their implications for disease trajectory and treatment response in adults remains limited.

Recent efforts to elucidate the co-occurrence of *BRAF* alterations with other genomic mutations and patient outcomes in adult gliomas are limited by case numbers or a particular focus on specific alterations, such as canonical *BRAF*^*V600E*^ mutations and *BRAF* amplifications^13–16^. Studies have not investigated the range of *BRAF* mutations, co-occurring alterations, and their clinical implications across a large set of sequenced genes, nor have there been robust comparisons of *BRAF* alteration types between adult and pediatric gliomas.

In this study, we retrospectively characterize a large multi-institutional cohort of adults with *BRAF*-mutated gliomas. We identify the clinical phenotypes, genomic signatures, and molecular features associated with different functional classes to assess prognostic implications and guide therapeutic approaches.

## Results

### Patient cohort

**Fig. 1:**
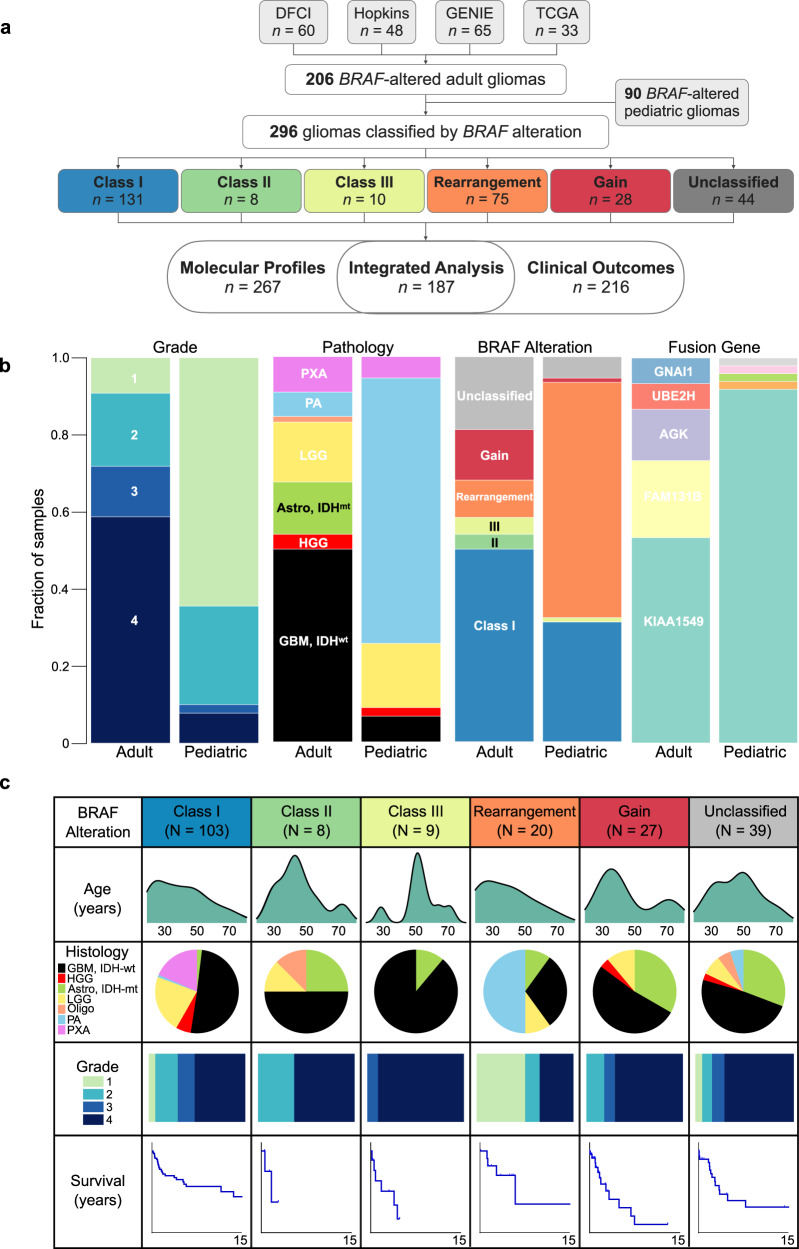
Characteristics of adults with BRAF-altered gliomas. **a** Study schematic demonstrating the source of cases and six cohorts into which *BRAF* alterations were categorized based on their known or presumed function. **b** Proportion of tumors with key pathological features among glioma samples from 206 adults and 90 pediatric patients. **c** Composition and clinical features of adult glioma classes by *BRAF* alteration including age at diagnosis, clinical histo-pathologic diagnosis, tumor grade, and overall survival (when known).

We obtained clinical and molecular data from 296 patients with *BRAF*-altered glioma (151 males, 145 females), including 206 adults (median 43 years, range 18–85 years) and 90 children (age <18 years, median 10 years, range 0–17 years). Subsets of this cohort had molecular (*n* = 267) and clinical outcomes (*n* = 216) data, with the majority of patients (*n* = 187) having both (Fig. 1a). Most samples were obtained at initial diagnosis (*n* = 220), with 56 from a follow-up resection or at an unknown time (*n* = 20).

The spectrum of histologic grades, pathology, and specific *BRAF* alterations in adults contrasted with that in children (Fig. 1b and Table 1). The most common histopathologic diagnosis in adults was glioblastoma (*n* = 108, 36%) (Table 1 and Fig. 1b, c). By contrast, low-grade gliomas, especially pilocytic astrocytomas, predominated in pediatric *BRAF*-altered cases (*p* = 0.0003). *BRAF* fusions and Class I mutations were most prevalent in children, while adult gliomas harbored a broad range of *BRAF* alterations, including oncogenic Class II/III alterations, copy number alterations, and otherwise unclassified alterations, all of which were rare in pediatric glioma.

**Table 1:** Summary of available clinical characteristics of adult and pediatric patients by *BRAF* alteration class.

	Class I		Class II		Class III		Rearrangement		Gain		Hypermutated		Unclassified	
Characteristic	Adult	Pediatric	Adult	Pediatric	Adult	Pediatric	Adult	Pediatric	Adult	Pediatric	Adult	Pediatric	Adult	Pediatric
Number of patients	103	28	8	0	9	1	20	55	27	1	13	1	26	4
Sex														
Male	44 (43%)	14 (50%)	3 (38%)		8 (89%)	1 (100%)	13 (65%)	33 (60%)	10 (37%)	1 (100%)	7 (54%)	1 (100%)	15 (58%)	1 (25%)
Female	59 (57%)	14 (50%)	5 (63%)		1 (11%)	0	7 (35%)	22 (40%)	17 (63%)	0	6 (46%)	0	11 (42%)	3 (75%)
Age at diagnosis^a^														
Median (range), years	39 (18–86)		43.5 (28–76)		54.5 (28–75)		36.5 (18–76)		41 (25–83.1)		46 (23.4–68)		50 (21–78)	
18–34	40 (39%)		2 (25%)		1 (11%)		9 (45%)		6 (22%)		2 (15%)		7 (27%)	
35–50	32 (31%)		4 (50%)		1 (11%)		6 (30%)		12 (44%)		6 (46%)		7 (27%)	
>50	31 (30%)		2 (25%)		7 (78%)		5 (25%)		9 (33%)		5 (38%)		12 (46%)	
Tumor histology														
Glioblastoma, *IDH* wildtype	52 (50%)	3 (11%)	4 (50%)		8 (89%)	0	6 (30%)	0	14 (52%)	1 (100%)	4 (31%)	1 (100%)	15 (58%)	1 (25%)
Other high grade glioma	6 (6%)	0	0		0	1 (100%)	0	0	1 (4%)	0	1 (8%)	0	0	1 (25%)
Astrocytoma, *IDH* mutant	2 (2%)	0	2 (25%)		1 (11%)	0	2 (10%)	0	9 (33%)	0	6 (46%)	0	6 (23%)	0
Low grade glioma	23 (22%)	14 (50%)	1 (13%)		0	0	2 (10%)	1 (2%)	3 (11%)	0	0	0	3 (12%)	0
Oligodendroglioma	0	0	1 (13%)		0	0	0	0	0	0	2 (15%)	0	0	0
Pilocytic astrocytoma	1 (1%)	7 (25%)	0		0	0	10 (50%)	53 (96%)	0	0	0	0	2 (8%)	2 (50%)
Pleomorphic xanthoastrocytoma	19 (18%)	4 (14%)	0		0	0	0	1 (2%)	0	0	0	0	0	0
Grade														
1	7 (7%)	5 (18%)	0		0	0	10 (50%)	51 (93%)	0	0	0	0	2 (8%)	2 (50%)
2	23 (22%)	19 (68%)	3 (38%)		0	0	3 (15%)	4 (7%)	5 (19%)	0	1 (8%)	0	3 (12%)	0
3	18 (17%)	1 (4%)	0		1 (11%)	1 (100%)	0	0	3 (11%)	0	3 (23%)	0	2 (8%)	0
4	55 (53%)	3 (11%)	5 (63%)		8 (89%)	0	7 (35%)	0	19 (70%)	1 (100%)	9 (69%)	1 (100%)	19 (73%)	2 (50%)
Survival, available *N*	72	16	5		9	1	13	38	25	1	10	1	22	3
Deceased	26 (36%)	0	2 (40%)		6 (67%)	1 (100%)	4 (31%)	2 (5%)	12 (48%)	1 (100%)	6 (60%)	0	10 (45%)	1 (33%)
Alive	46 (64%)	16 (100%)	3 (60%)		3 (33%)	0	9 (69%)	36 (95%)	13 (52%)	0	4 (40%)	1 (100%)	12 (55%)	2 (67%)
Median OS (95% CI), months	164.5 (62.6–NA)	NR	20.1 (7.2–NA)	NA	46.8 (9.7–NA)	46.0 (NA)	71.1 (33.4–NA)	NR	38.8 (19.8–NA)	4.8 (NA)	58.6 (27.1–NA)	NR	41.4 (32.8–NA)	NR (20.2–NA)

Number of patients (%) denoted per BRAF alteration type unless otherwise specified.

*OS* overall survival, *NA* not applicable, *NR* not reached.

^a^All pediatric patients <18 years of age.

### Landscape of *BRAF* alterations in adult glioma

**Fig. 2:**
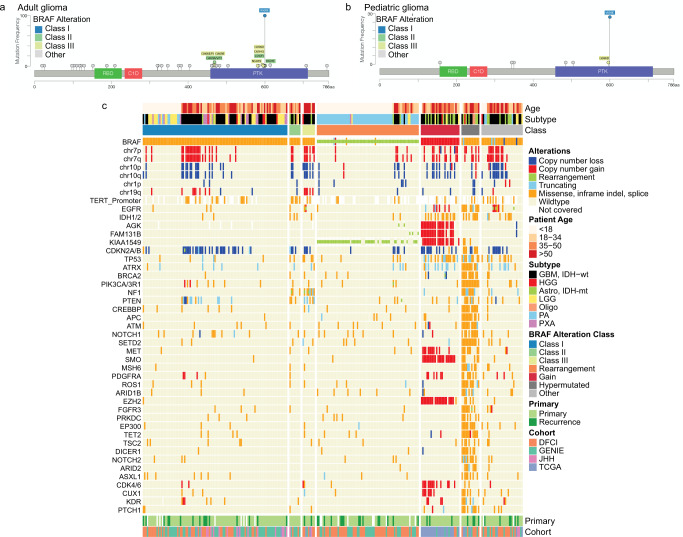
Genomic landscape of adult and pediatric BRAF-altered gliomas. Lollipop plot depicting the location of *BRAF* single-nucleotide variants (SNVs) identified in the **a** adult or **b** pediatric glioma cohort, with color denoting alteration class. **c** Co-mutation plot grouped by *BRAF* alteration class for all samples with genomic profiling data.

We explored the distribution of *BRAF* alterations across age and histopathologic groups, along with associations with other common alterations (*IDH1/2, EGFR, TERT, ATRX, H3K27M, CDKN2A/B, NF1, KRAS, HRAS*). While the most common SNV in *BRAF* was at p.V600E in both adult and pediatric gliomas, alterations at other sites known to affect *BRAF* dimerization or increase ERK signaling (Class II/III) were more common in adult samples (Fig. 2a, b). In addition to the previously described Class II and III alterations^3,4^, we observed a small number of alterations in close proximity to those nucleotides which likely occur in functional domains but have not yet been validated (within kinase domain: Q461E, I463M, V471I, G474R, V482G, L584I, S614P, S614P; within receptor-binding domain: R188K, G209S). Other alterations distant from the kinase domain may be functionally insignificant and incidental. For this analysis, samples were included in the *BRAF* Class II and III cohorts only if the SNV was previously shown to be ERK-activating^1^.

Approximately half of adults in our cohort had Class I *BRAF* alterations (*n* = 103/206; median age at diagnosis 39 years, range 18–86 years; 57% female). GBM was the most common histopathologic diagnosis (51%, *n* = 52), followed by low-grade glioma (22%), and PXA (18%), among others (Table 1). Overall survival across adults with Class I alterations was 165 months (Fig. 1c). In total, 2% (*n* = 2) had mutations in *IDH1/2*, 28% (*n* = 29) had loss of *CDKN2A/B*, 3.9% (*n* = 4) had alterations in *ATRX*, none had alterations in *H3K27* or *NF1*. We found 24% of *BRAF*^*V600E*^-mutant GBMs (*n* = 10/41) had mutations in the *TERT* promoter, 22% (*n* = 9) had chromosome 7p gain and 10q loss, and none had *EGFR* amplification. A subset of gliomas across all ages with Class I *BRAF* mutations harbored no other alterations, including a subset histologically defined as GBMs (Fig. 2c).

Class II alterations (3.9%; median age 44 years, range 28–76 years; Figs. 1c and 2c) were seen in eight adult and no pediatric patients. The alterations occurred at three amino acids (p.L597R, p.G469R/V/A, and p.K601E) previously described in melanoma^3^. Histopathology was predominantly GBM, though there were four grade 2 gliomas as well—one diffuse astrocytoma (*IDH*-WT), one oligodendroglioma (*IDH*-mutant, 1p/19q co-deleted), and two astrocytomas, *IDH*-mutant. Median survival was 20 months.

While Class II alterations are not dependent on RAS activation, we did observe *NF1* alterations in three specimens (38%). Of note, three specimens had concurrent *IDH1/2* mutations, two of which were known oncogenic alterations (Fig. 2).

Nine (4.3%; median age 55 years, range 28–76 years) patients in our adult cohort had Class III alterations (Fig. 1c and Table 1). These included p.G466R/E, p.G469E, p.N581S, p.D594G/N, and p.G596D. All but one tumor with Class III *BRAF* alterations were glioblastomas, while the remaining one was a grade 3 *IDH*-mutant astrocytoma. Median survival was 47 months. One pediatric patient with a grade 3 HGG harbored a Class III alteration.

Given their dependence on RAS activity, Class III alterations commonly co-occur with RTK alterations, *NF1* loss, or RAS-activating alterations. We observed mutations in RTK, RAS, or *ERK* pathway components in all adults with Class III altered gliomas (*n* = 9). Specifically, seven had *NF1* alterations, two had activating *EGFR* alterations, and one had an alteration in *MET*. Additionally, two had *TERT*-promoter mutations, four had *CDKN2A/B* losses, and one had an *IDH1/2* alteration (Fig. 2).

*BRAF* rearrangements are common in pediatric LGG, particularly pilocytic astrocytoma, and linked with favorable survival^7^. Twenty adult patients (9.7%; median age 42 years; range 18–76 years) had tumors harboring *BRAF* rearrangements (Fig. 1c and Table 1). Of these, 10 (50%) were PA, 6 (30%) were GBM, 2 (10%) were low-grade glioma, and 2 (10%) were astrocytoma, *IDH*-mutant (one grade 2 and one grade 4). The most common *BRAF* rearrangement partner was *KIAA1549* (*n* = 11) (Fig. 1b). Two *BRAF*-rearranged gliomas had *TERT* promoter mutations and five had loss of *CDKN2A/B*, all of which were grade 4. Median survival in the entire cohort was 71 months; 33 months in patients with GBM and not reached in those with PA.

In the pediatric cohort, *BRAF* rearrangements dominated (*n* = 55; 61%). The majority of rearrangements were *KIAA1549*-*BRAF* rearrangements (*n* = 47; 85%), with only one tumor each harboring *BCAS1, CCDC6, GIT2*, or *PTPRZ11* as a fusion partner (Fig. 1b). Clinically, all but two (1.8%) patients were diagnosed with a pilocytic astrocytoma, with the remaining being grade 2 PXA or LGG (Supplementary Fig. 1).

Twenty-seven adults had tumors with *BRAF* copy number gains. Age at diagnosis was bimodal with a peak at 35 years and second peak at 75 years. The majority of patients (70%, *n* = 19) had glioblastoma, while 9 tumors (33%) had *IDH* mutations. Median survival in this cohort was 39 months. Only one pediatric patient had a *BRAF* gain; histopathologic diagnosis was GBM, *IDH*-WT and clinical deterioration was rapid.

Tumors with unclassified *BRAF* alterations comprised 19% of the adult cohort (*n* = 39) and 6% of pediatric cases (*n* = 5) (Figs. 1b and 2c). In adults, median age at diagnosis was 49 years (range 21–78). Seventeen (44%) were female. The majority of alterations in this cohort were SNVs (*n* = 30, 77%), with 4 intragenic rearrangements, 2 having focal loss of BRAF, and 3 damaging mutations (Fig. 2c). Median overall survival in this cohort was 42 months.

Interestingly, a subset of unclassified adult tumors (*n* = 13, 6.3%) had increased tumor mutation burden across the genome, consistent with a hypermutated phenotype (Fig. 2c). Three hypermutated tumors had known pathogenic *IDH* mutations, while four had *IDH* mutations of unknown significance. Nine were newly diagnosed gliomas and 4 were recurrent tumors. Of four patients with detailed clinical treatment data, all received temozolomide prior to the surgical specimen for which NGS was obtained.

### Association of molecular alterations with *BRAF* class

**Fig. 3:**
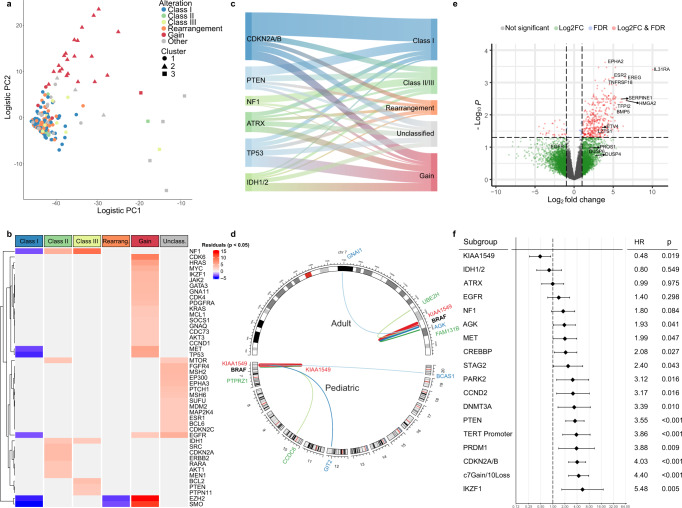
Co-occurring mutations by BRAF alteration. **a** Logistic PCA plot with k-means clustering of *BRAF*-altered gliomas based on genomic alterations. **b** Gene alterations significantly enriched or depleted across *BRAF*-altered glioma classes, shaded by chi-square residuals on post hoc analysis where red and blue signify positive and negative relationships, respectively. **c** Top co-occurring gene mutations by *BRAF* alteration class, colored bar thickness represents number of cases. **d** Structural DNA rearrangements in 20 adult gliomas and 55 pediatric gliomas. Lines connect the chromosomal locations of *BRAF* and fusion gene partners, line thickness is proportional to rearrangement frequency in study cohort. **e** Differentially expressed genes in *BRAF* Class I tumors versus other *BRAF*-altered gliomas in transcript sequence data from TCGA samples. **f** Univariate Cox proportional hazards analysis evaluating associations between molecular alterations and overall survival in 187 adult glioma patients. Hazard ratios (diamonds) and 95% confidence intervals (horizontal lines) are depicted.

We next evaluated associations between different *BRAF* alteration classes and other molecular characteristics. We performed unsupervised clustering using genomic data, which identified 3 clusters. Gliomas with *BRAF* gains (Cluster 2) were more closely associated with one another than gliomas with *BRAF* Class I–III mutations or fusions (Fig. 3a and Supplementary Table 1). A small cluster (Cluster 3) of primarily high-grade gliomas contained mostly unclassified *BRAF* alterations. Gliomas with *BRAF* gains commonly had concomitant gains in *MET, SMO, EZH2*, and *CDK6* (*p* < 0.001 each; Supplementary Table 2), along with other genes on 7q, which may reflect larger chromosomal duplications (Fig. 3b). Interestingly, *HRAS*, *MYC*, and *TP53* alterations were also enriched in the gains cohort (*p* < 0.00001).

In *BRAF*^*V600E*^-altered gliomas, we found no significant co-occurring oncogenes when compared with other alteration classes. However, Class I alterations were negatively correlated with alterations in *NF1, EGFR, TP53*, and *MET* (Fig. 3b, c). As anticipated, concurrent alterations in RAS pathway components were rarely observed (Fig. 2c).

Several other correlations were observed between classes, some anticipated based on known activity (Fig. 3b, c and Supplementary Table 2). Tumors with Class II and III alterations were enriched for *NF1* alterations (Fig. 3a; *p* < 0.00001). *EGFR* and *FGFR4* alterations were associated with unclassified *BRAF* alterations after excluding hypermutated samples (*p* = 0.0003). Notably, *CDKN2A/B* loss, *ATRX*, and *PTEN* mutations were observed across all groups.

*BRAF* rearrangements were not associated with other molecular alterations in our panel. Within the *BRAF* rearranged cases, non-*KIAA1549* rearrangements were associated with higher pathologic grade. Of note, all rearrangements in adult glioma were intrachromosomal rearrangements on the long arm of chromosome 7, while in pediatric glioma several interchromosomal rearrangements were observed (Fig. 3d).

To further explore differences between tumors with distinct *BRAF* alterations, we analyzed RNA sequencing data from 18 TCGA samples in our cohort. These tumors harbored Class I alterations (*n* = 4), *BRAF* gains (*n* = 10), or unclassified alterations (*n* = 4). Compared to other *BRAF*-altered gliomas, tumors with Class I alterations had elevated expression of several well-known tumor microenvironment marker genes (Fig. 3e). Notably, gliomas with Class I alterations were enriched for transcripts associated with MEK functional activation (e.g., *ETV4*, *LZTS1*), which can also be markers of MEK inhibitor sensitivity^17,18^. They also demonstrated increased expression of *EREG* (an *EGFR* ligand) and the *EPHA2* receptor, which are associated with resistance to treatment with *EGFR* or *BRAF* inhibitors^19,20^. This finding is consistent with recent observations that *BRAF*-mutant GBM have distinct expression profiles compared with non-*BRAF*-mutant GBM^16^. Meanwhile, tumors with *BRAF* gains were enriched for genes associated with transcriptional regulation compared to other samples (Supplementary Fig. 2).

### Impact of *BRAF* class on clinical outcome

We evaluated the impact of clinical and molecular features on outcomes in this retrospective cohort of 187 patients (127 adults, 60 children) with combined data. Diagnosis of pilocytic astrocytoma, oligodendroglioma, and younger age at diagnosis were associated with more favorable prognoses, while age >50 years, GBM pathology, *IDH*-WT status, and higher tumor grade were associated with inferior prognoses (*p* < 0.001) (Fig. 3f).

Molecular features such as *CDKN2A/B* loss, *PTEN*, and *TERT* promoter mutations were associated with inferior prognoses (*p* < 0.001). Other alterations associated with worse prognosis across pediatric and adult patients included alterations in *AGK, MET, CREBBP, STAG2, PARK2, CCND2, DNMT3A, PRDM1*, and *IKZF1* (*p* < 0.05; Fig. 3f). Some alterations associated with worse prognosis (*PTEN*, *EGFR*, *NF1*) correlated with increased age (*p* < 0.0005; Supplementary Table 2). The only alteration associated with improved survival was *KIAA1549*, as expected given its association with pilocytic astrocytoma.

**Fig. 4:**
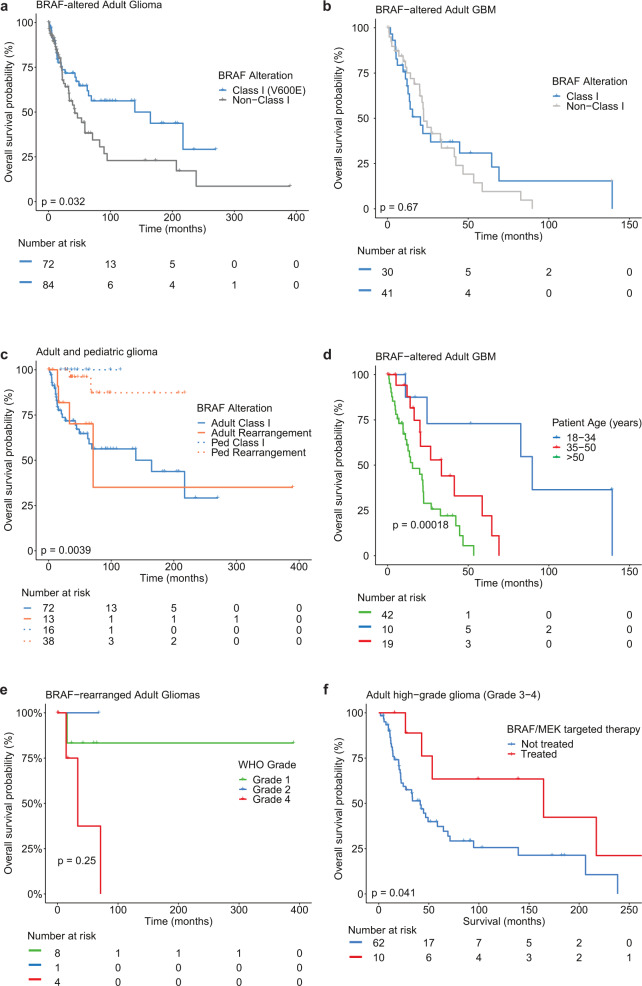
Overall survival in patient subgroups with BRAF-altered glioma. Survival of adults with *BRAF* V600E alterations (blue) versus all other alterations (gray) for **a** all glioma or **b** glioblastoma only. **c** Overall survival for adult and pediatric patients with gliomas harboring Class I alterations or *BRAF* rearrangements separated by age and alteration type. **d** Overall survival of patients with glioblastoma by age categories. **e** Survival in adults with *BRAF* rearrangements separated by tumor grade. **f** Overall survival of adults with high-grade glioma treated (blue) or not treated (red) with targeted therapy.

Gliomas with distinct *BRAF* alteration types exhibited varying survival (Fig. 1c). We found that overall survival was significantly prolonged in adults with Class I alterations compared to other alterations (*p* = 0.032; Fig. 4a). This appears to be partially driven by grade, as no survival difference was observed between GBM patients with Class I *BRAF* alterations and those with non-Class I alterations (*p* = 0.67, Fig. 4b), with median survival of 22 months across patients with GBM. This analysis did not control for the type or number of lines of therapy.

Notably, our cohort demonstrated a significant difference in survival between pediatric and adult patients with either *BRAF* Class I alterations or rearrangements (*p* = 0.0012; Fig. 4c), underscoring the large difference in prognosis between adult and pediatric tumors, even with the same molecular alteration. Younger age remained associated with improved survival in 71 adult GBMs with survival data (*p* < 0.0001, Fig. 4d).

Additionally, we observed similar survival between adult patients with Class I alterations and rearrangements (Fig. 4c). When adults with rearrangements were separated by grade, there was a survival difference between grade 1 and grade 4 gliomas, suggesting grade serves as a more accurate prognostic indicator than the presence of a *BRAF* rearrangement alone (Fig. 4e). In adults with grade 3–4 astrocytoma, BRAF/MEK-targeted therapy was associated with improved overall survival (Fig. 4f) compared with grade 3–4 patients who did not receive targeted therapy, suggesting targeted therapy has the ability to impact disease trajectory.

### Effect of targeted therapy in adults with *BRAF* mutant glioma

**Fig. 5:**
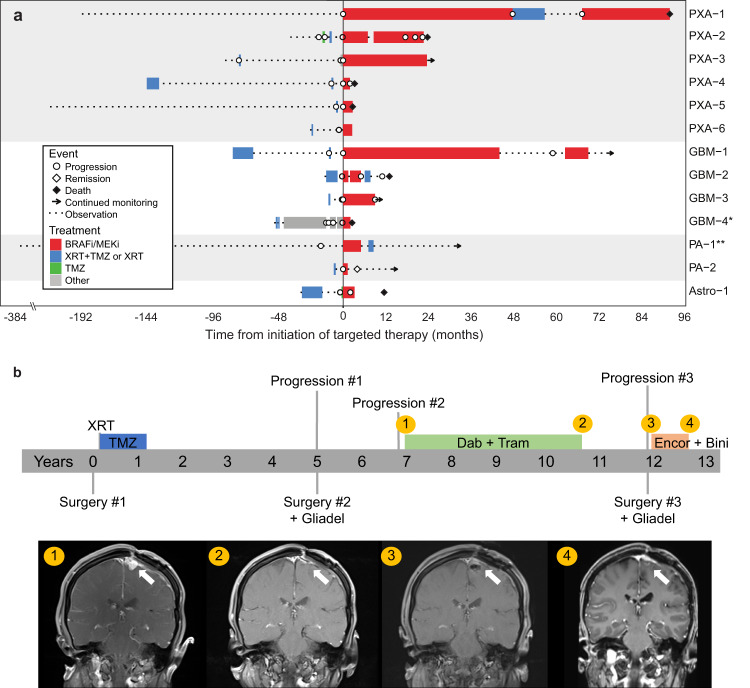
Treatment response in patients with BRAF-altered glioma. **a** Swimmers plot depicting clinical course of patients indicating time on *BRAF*-targeted therapy (red), radiation alone or radiation combined with temozolomide (blue), temozolomide treatment alone (green), other anti-neoplastic treatment (gray), or observation (dashed line). Time of events are noted for progression (open circle), remission (open diamond), death (black diamond), or continued treatment and/or observation (arrow). All patients are centered on the time they initiated targeted therapy, with lines to the left of midline depicting clinical course prior to targeted therapy. All patients had a BRAF V600E mutation on molecular analysis, with the exceptions of patients “GBM-4” and “PA-1”, whose gliomas had a BRAF Class III (N581S) mutation and *KIAA1549*-*BRAF* fusion, respectively. **b** Graphic depiction of clinical course in a patient with epithelioid GBM treated with multiple lines of *BRAF*-targeted therapy.

Thirteen adults with recurrent gliomas received BRAF/MEK-targeted therapy (Fig. 5a), two of whom have been previously described^21,22^. Eleven had Class I mutations and 2 had non-Class I alterations. Median age at diagnosis was 26 years and 38% (*n* = 5) were female. Targeted therapy consisted of BRAF inhibition (vemurafenib, *n* = 3; vemurafenib followed by dabrafenib, *n* = 1), MEK inhibition (trametinib, *n* = 1), or a combination (dabrafenib and trametinib, *n* = 8). BRAF and/or MEK inhibition was first-line therapy in two patients. However, the majority of patients had BRAF and/or MEK inhibition either initiated as second-line therapy following radiation with or without concurrent temozolomide (*n* = 5, 38%), or initiated after multiple lines of therapy (*n* = 6, 46%). Six patients had stable disease for four or more months on targeted therapy. Median time to progression while on targeted therapy was 5.0 months. Overall survival for all patients receiving targeted therapy was 165 months and 53 months for patients with GBM. Four patients (31%) in the cohort were continuing targeted therapy at the time of last follow-up. Reasons for discontinuation of therapy were treatment-related side effects (*n* = 4), radiological evidence of progression (*n* = 2), death (*n* = 2), or unknown (*n* = 1).

The complexities of *BRAF* targeted therapy are highlighted by one patient whose case has been partially described previously (Fig. 5b)^21^. She was diagnosed at 23 years of age with epithelioid glioblastoma and treated with radiation and concurrent temozolomide, followed by 12 cycles of adjuvant temozolomide. Her cancer recurred 5 years after diagnosis, at which time she underwent repeat resection with carmustine wafer placement. Two years later, she had a second recurrence and started dabrafenib plus trametinib for 44 months before discontinuing therapy due to neutropenia. She was monitored clinically with no evidence of recurrence for 14 months, before another radiographic recurrence prompted repeat resection, carmustine wafer placement, and initiation of encorafenib with binimetinib. She remained on therapy for 6 months before discontinuation due to colitis and then remained disease-free for another 6 months before recurrence.

## Discussion

While *BRAF* alterations are significant and potentially targetable in glioma, the frequency, spectrum, and clinical implications of *BRAF* alterations in adult glioma have not been previously characterized due to their rarity. In comparison to prior studies, this multi-institutional cohort has improved coverage of clinical outcomes and NGS data. This allowed characterization of non-canonical mutations in *BRAF* present in adult gliomas; improved estimates of relative frequencies and composition of *BRAF* alteration types; and integrated clinical-genomic analyses to potentially aid in prognostication.

In this cohort, several important characteristics of *BRAF* alterations in adult glioma emerge. First, *BRAF* alterations co-occur with alterations similar to those observed in other cancers, suggesting they may portend similar functional effects in glioma. Second, while overall survival in all patients with Class I alterations was improved relative to other classes, there was no improved survival in GBM. Third, the spectrum of *BRAF* alterations is different between pediatric and adult glioma, with important ramifications for clinical outcomes. Lastly, BRAF-targeted therapy can benefit a subset of patients with targetable *BRAF* alterations.

As in other cancers, most Class I *BRAF* alterations occurred in the relative absence of additional oncogenic alterations, suggesting they are the primary oncogenic drivers in these tumors. We also observed a transcriptional signature of ERK-dependence in tumors with Class I alterations^18,23^, suggesting potential sensitivity for targeted therapy. Concurrent alterations in other RAS pathway members (*NF1*, *EGFR*, etc.) occurred in Class II/III tumors, consistent with the proposed mechanism of ERK activation, while also suggesting ERK-dependence is the primary oncogenic driver in these cancers^3,4^. Interestingly, gliomas with *BRAF* gains often exhibited focal chromosome 7q gains, suggesting either an advantageous amplification or that *BRAF* is a passenger amongst other genes promoting glioma progression^24^. Of note, 14 tumors in our cohort had hypermutated phenotypes, most commonly with unclassified *BRAF* alterations. A subset of hypermutated gliomas (23%) had *IDH*-mutations, which have a known predilection toward hypermutation following temozolomide chemotherapy^25^. *BRAF* alterations in hypermutated tumors may be passenger alterations or, as observed recently, may confer a growth advantage^26^.

Associations between *BRAF* alteration class and outcome in adults were mixed. Across all patients with Class I alterations, we observed improved overall survival compared to other classes. However, there was no survival advantage among GBM patients with Class I alterations compared to other patients with GBM, suggesting the transition to GBM allows cells to compensate for any growth disadvantage of *BRAF*-driven ERK dependence^27^. This phenomenon was also demonstrated in patients with *BRAF* fusions. While *BRAF* fusions are associated with an indolent course in pediatric patients, in adults there was a bimodal survival distribution of pilocytic astrocytomas and other higher-grade pathologies with a more aggressive clinical course. This observation underscores the importance of pathologic grade and other clinical features rather than *BRAF*-alteration alone for prognostication^28–31^. Age, *CDKN2A/B* loss, and molecular markers of GBM were correlated with a worse prognosis, regardless of *BRAF* alteration, emphasizing their importance for guiding the timing and type of treatment^29^. Given the retrospective nature of our cohort, most patients did not receive targeted therapy, so it is unclear whether *BRAF-*targeted therapy has changed the outcomes of patients with GBM^11,12^. Another nuance to the role of *BRAF* alterations in glioma prognosis is the broad range of alterations present in adult glioma. Further mechanistic work is necessary to understand whether the *BRAF* classes observed imply functional pathway dependency and whether they could be exploited with specific therapeutic strategies.

Identification of a targetable *BRAF* alteration provides patients with novel therapeutic opportunities. In our small retrospective cohort of patients treated with targeted therapy, we observed clinical benefit in a subset of patients. In prospective clinical trials, response rates of 70% and 33% have been observed among adults with low or high grade glioma, respectively^11,12^. Clinical trials of dimer-disrupting BRAF inhibitors with potential efficacy in additional *BRAF*-altered classes are ongoing in adults with glioma (NCT05503797). Additionally, improved blood brain barrier penetration may provide additional benefit to patients with central nervous system involvement (NCT04543188). Given the growing list of therapeutic options, accurate identification of BRAF alterations is critical for patient care. Molecular profiling has proven value for accurate tumor diagnoses and is becoming widespread in adults with brain tumors^29^. In cases where a tissue sample is not feasible, liquid biopsy shows promise to accurately identify targetable alterations such as BRAF from plasma samples^32,33^.

This landscape of *BRAF* alterations in adult glioma provides an invaluable resource to clinicians evaluating the functional implications of various *BRAF* alterations in adults with glioma. Further work is necessary to prospectively validate outcomes and identify the role of other *BRAF* alterations in patients with glioma. In patients with targetable alterations (Class I–III), small molecule inhibitors can have a significant clinical benefit, underscoring the importance of accurately identifying and classifying *BRAF* alterations in patients of all ages with glioma.

## Methods

### Patient data collection

Patients aged ≥18 years at the time of diagnosis with a glioma containing a *BRAF* alteration on sequencing were identified at the Dana-Farber/Harvard Cancer Center (DFCI), Johns Hopkins Hospital (JHH), and two public repositories: The Cancer Genome Atlas Program (TCGA) and Project Genomics Evidence Neoplasia Information Exchange (GENIE). A single glioma tissue sample collected between 2008–2020 was included per patient (either primary resection or recurrence). For comparison, 90 pediatric (<18 years old) patients with gliomas harboring a *BRAF* alteration in sequencing data were identified from DFCI, TCGA, and GENIE, with demographic data available for a subset (*n* = 57). Clinical, histopathologic, and molecular data from tumor tissue were collected and analyzed retrospectively. Medical record review for clinical characteristics and outcomes was performed under protocols approved by the institutional review boards of DFCI and JHH. A waiver of consent was obtained for this retrospective study at Johns Hopkins given infeasibility of consent since many patients were deceased (IRB00243637). Written informed patient consent was obtained for all patients under Dana-Farber Cancer Institute IRB protocol 10–417.

### Clinical and pathologic annotation

Pathological diagnosis of tumors within this study was based on the 2021 WHO classification of CNS tumors to the extent possible; however, many cases had insufficient information for precise classification using this system and some broader categories were also used^29^. Available information on tumor pathologies were reviewed and samples were grouped into seven general pathological categories for further analysis by a neuropathologist (CGE). These categories included glioblastoma, *IDH*-wildtype (GBM, *IDH*-WT); astrocytoma, *IDH*-mutant (Astro, *IDH*-mt, Grade 2–4); oligodendroglioma (Oligo, *IDH*-mt, Grade 2–3); pleomorphic xanthoastrocytoma (PXA, Grade 2–3); and pilocytic astrocytoma (PA). A subset of tumors did not fall into one of those diagnostic categories and were grouped based on their grade: other high-grade glioma (HGG; Grade 3–4) and other low-grade glioma (LGG; Grade 1–2). Overall survival (OS) was obtained from public databases or calculated from electronic medical records as time from radiographic diagnosis to date of death. Records from 13 adult patients treated with *BRAF*-targeted therapy were further reviewed to determine treatment type(s), duration, response, and time to progression(s).

We ran an optimal cut-point analysis using maximally selected rank statistics to identify any age inflection points that significantly corresponded with survival in our overall cohort and found inflection points at 34 and 51 years (Supplementary Fig. 3). Consequently, for the purposes of this study, we defined age interval groups as <18, 18–34, 35–50, and >50 years of age.

### Genomic characterization

Genomic mutation profiles were evaluated from panel-based next-generation sequencing assays—which included 227–447 cancer-associated genes (OncoPanel, versions 1–3)^34,35^ for DFCI samples and 27–435 genes (Neuropath NGS Panel and Solid Tumor Panel versions 2–4)^36^ for JHH samples—as well as from publicly available targeted mutational profiling microarray sequencing data for 341–398 genes (MSK-IMPACT)^31^ and exome sequencing data covering 1.06 Mb of cancer-associated genes^37^ for GENIE and TCGA samples, respectively. Available mutation data were compiled, including single-nucleotide variants (SNV), copy number variants (CNV), and structural rearrangements. In genomic analyses, we incorporated missense mutations, in-frame insertion-deletions (indels), splice site mutations, “truncating” mutations (nonsense mutations, frameshift indels), high-level copy number amplifications^38^, high-level copy number losses, and structural rearrangements such as gene-gene fusions. Low-copy gains, single-copy deletions, synonymous mutations, and intronic variants were excluded. Genes covered in at least two targeted panels and altered in at least 5% of patients in the combined dataset were included in downstream analysis.

*BRAF* alterations were classified into six groups based on previously defined functional classes. We used Class I to designate *BRAF*^V600^ alterations that activate *ERK* signaling and are oncogenic as monomers^2^. Class II and Class III *BRAF* alterations activate *ERK* signaling through dimerization that is independent or dependent on upstream *RAS* activation, respectively^3,4^. Known pathogenic *BRAF* rearrangements (“fusions”) and *BRAF* gene amplifications (“gains”) were also classified into separate categories, with all remaining alterations categorized as “unclassified”.

We parsed hypermutated samples into a distinct category based on total mutational count cutoffs. Tumor mutational burden (TMB) was estimated from targeted sequencing data as the total number of nonsynonymous mutations and indels across the coding regions covered by the gene panels (coverage range between 0.7–1.3 Mb). For downstream analyses, hypermutated samples were defined as those with greater than the 95th percentile TMB (>55 total nonsynonymous mutations).

### Statistical analyses

All statistical analyses were performed using R statistical software, version 3.6.3 (R Foundation for Statistical Computing; www.r-project.org). Clustering analysis was performed using 136 genes with cross-coverage across all non-hypermutated samples with genomic data. For each sample, genes were binarized as either altered or non-altered. Logistic principal component analysis (PCA) was implemented using the logisticPCA package (CRAN) to reduce the dimensionality of the binary alterations matrix (genes x samples) and k-means clustering was performed on the first 25 logistic principal components. We used the silhouette and elbow methods to determine the optimal number of k-means clusters.

Correlations between *BRAF* alteration types, patient characteristics, and other genomic alterations (binarized as present or absent for each gene) were evaluated using Chi-squared test constructed from pairwise contingency tables. Strength of association was assessed using Cramer’s V statistic. Post hoc analysis was applied on significant associations to dissect directions of effect.

Associations between clinical, histopathologic, and molecular covariates of interest and overall survival were assessed by univariate analysis using Cox proportional hazards models. Correction for multiple comparisons was performed using the Benjamini–Hochberg method. Variables with an adjusted false discovery rate (FDR) < 0.05 and altered in at least five individual patients were reported as significant. Overall survival rates were compared by the Kaplan–Meier method with censoring to date of last follow-up and significance determined by log-rank test. All survival analyses were performed using the survival (CRAN) and survminer (CRAN) packages.

### RNA sequencing analysis

HTSeq counts matrices^39^ available for TCGA samples in the cohort were obtained from the public glioblastoma (TCGA-GBM) and low-grade glioma (TCGA-LGG) data repositories. Differential expression analysis was performed using quasi-likelihood *F*-tests after standard filtering, normalization, and dispersion estimation steps in EdgeR. Pathway analyses were conducted using goseq. Volcano plot and treemap figures were constructed using the EnhancedVolcano and rrvgo R packages. The Benjamini–Hochberg method was used to account for multiple hypothesis testing in both differential expression and pathway analysis.

### Reporting summary

Further information on research design is available in the Nature Research Reporting Summary linked to this article.

## Supplementary information

Supplemental Information

REPORTING SUMMARY

## Data Availability

Datasets from TCGA used in this project are publicly available using the National Cancer Institute Genomic Data Commons (GDC) Data Portal (https://portal.gdc.cancer.gov/). TCGA data are listed under the Project IDs “TCGA-LGG” and “TCGA-GBM.” Datasets from GENIE used in this project are publicly available using the cBioPortal (https://genie.cbioportal.org/) from the “GENIE Cohort v12.0-public” study. Consent for public data sharing was not obtained from DFCI and JHH patients, so datasets from DFCI and Johns Hopkins Hospital are available upon data usage agreement from the corresponding authors.

## Code availability

Code used for this analysis is available at https://github.com/pclangat/braf-glioma.
