# Supplementary material for: Integrated molecular and clinical analysis of BRAF-mutant glioma in adults
Source: NPJ Precis Oncol. 2023 Feb 28;7:23. doi: 10.1038/s41698-023-00359-y (PMC9975216; doi:10.1038/s41698-023-00359-y)
Supplement: Supplementary file 1 — Supplemental Information [file 41698_2023_359_MOESM1_ESM.pdf]

Supplementary Figures & Tables

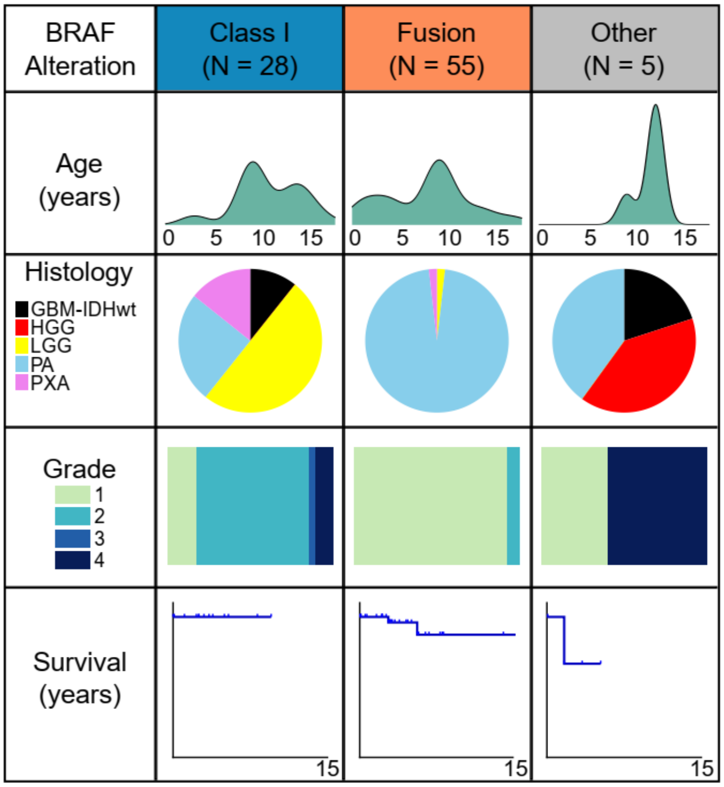

Supplemental Figure 1. Composition and clinical features of pediatric gliomas by BRAF alteration.

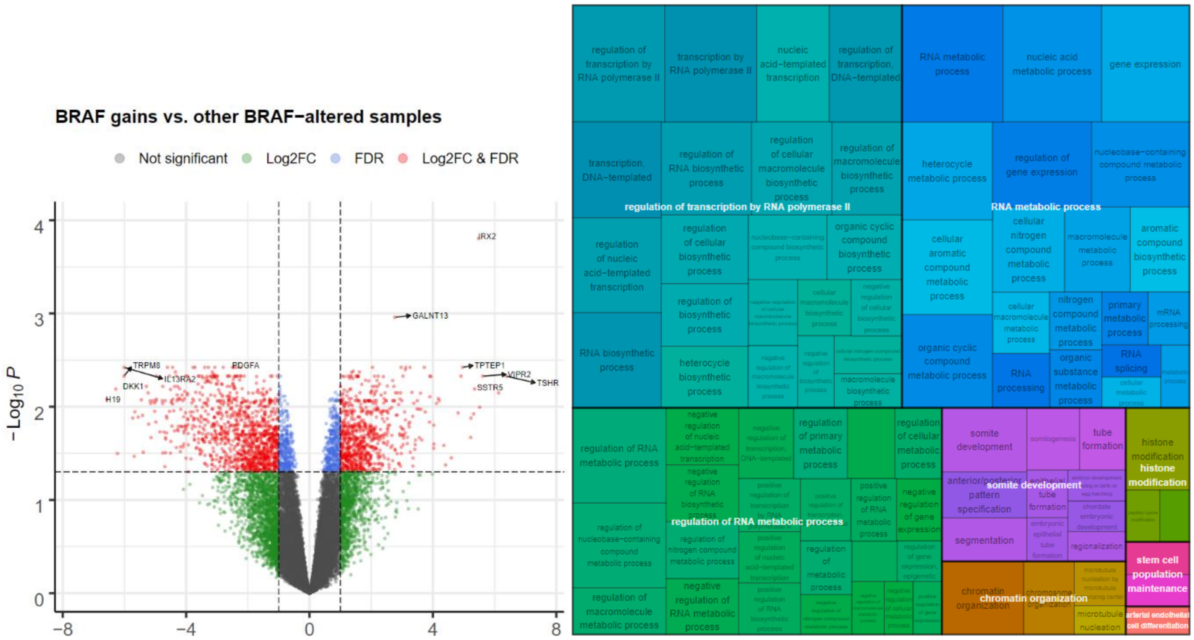

Supplemental Figure 2. A) Volcano plot and B) gene ontology biological pathway analysis of genes enriched in tumors with *BRAF* gains compared to all other *BRAF* alteration classes.

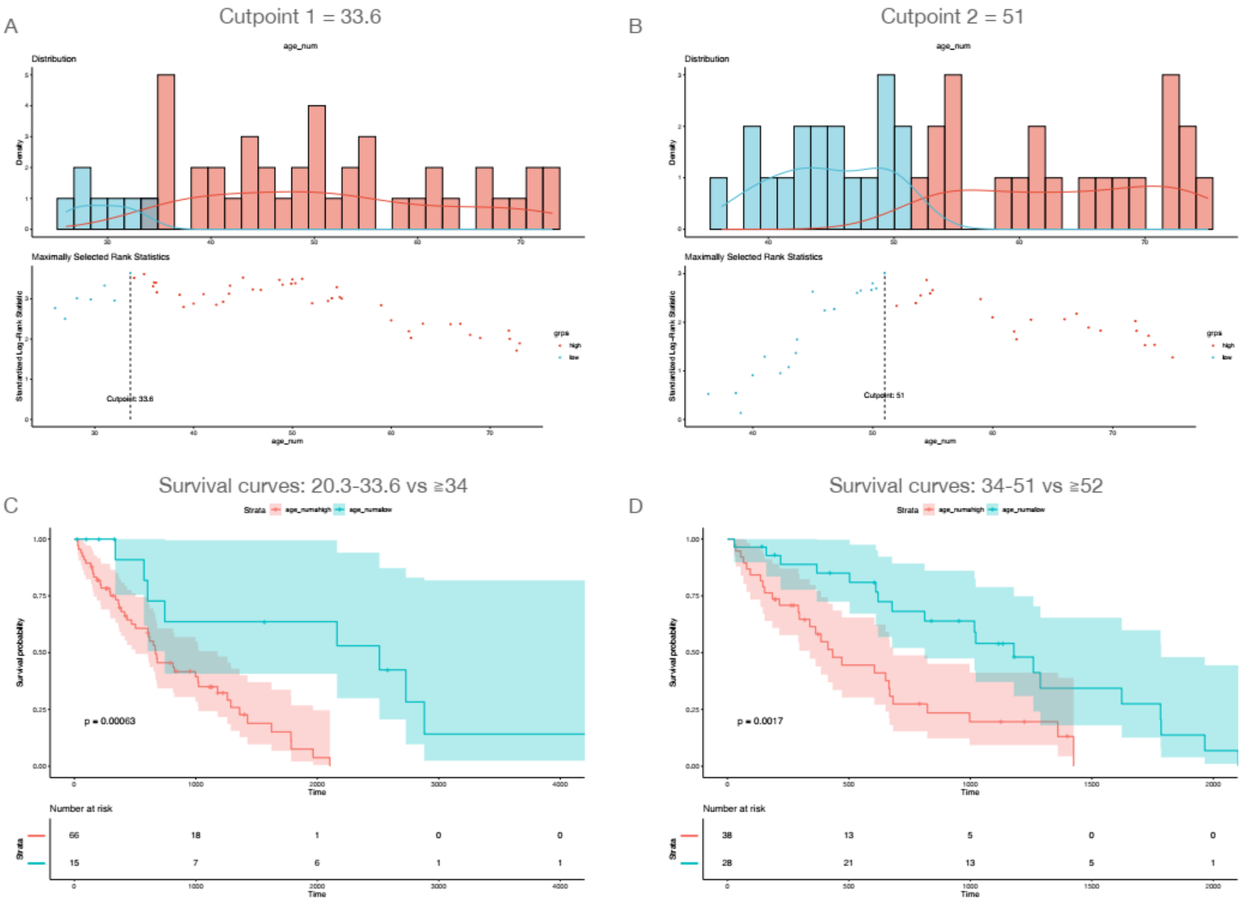

Supplemental Figure 3. Cutpoint analysis based on overall survival for determination of optimal age intervals for adult patients of this cohort. A,C) Cutpoint 1 and B,D) each demonstrated a significant inflection point. Based on rounding of cutpoint results to nearest 5 years, the proposed age categories used in this study are: Young Adult, 18-34 years (n=15); Middle, 35-50 years (n=28); Older, >50 years (n=38).

Supplemental Table 1. Clinical and molecular features of BRAF sample k-means clusters.

		% in Cluster 1 (n = 225)	% in Cluster 2 (n = 25)	% in Cluster 3 (n = 7)
Clinical	WHO I	31.6	0	0
	WHO II	23.6	16	14.3
	WHO III	7.56	16	0
	WHO IV	37.3	68	85.7
	Primary	81	92	71.4
	Recurrent	19	8	28.6
	Glioblastoma	34.2	48	42.9
	Oligodendroglioma	0.444	0	0
	Pleomorphic xanthroastrocytoma	8	0	0
	Pilocytic astrocytoma	32.4	0	0
	Astrocytoma, IDH-mutant	4.89	40	57.1
	Other high grade glioma	3.11	4	0
	Other low grade glioma	16.9	8	0
	<18 years old	39.6	0	0
	18-34 years old	19.1	20	0
	35-50 years old	18.2	44	71.4
	50+ years old	23.1	36	28.6
	Female	47.1	56	42.9
	Male	52.9	44	57.1
BRAF alteration type	Class I	46.7	0	0
	Class II	3.11	0	14.3
	Class III	3.56	0	14.3
	Fusion	32.9	0	0
	Gain	2.22	88	14.3
	Other	11.6	12	57.1
Other altered genes	IDH1/2	5.41	52	57.1
	MGMT methylation	50	80	100
	TERT	13.8	8	14.3
	SMO	1.33	84	42.9
	EZH2	1.78	80	14.3
	ROS1	3.11	0	71.4
	TSC1	1.33	12	71.4
	ALK	1.78	4	71.4
	EGFR	4.89	32	71.4
	TP53	9.78	68	71.4
	ESR1	0	0	57.1
	MSH2	1.78	0	57.1
	PIK3CA	6.67	0	57.1
	SMARCA4	0.444	8	57.1

MSH6	0.889	4	57.1
ABL1	1.33	8	57.1
MET	2.67	40	57.1
CREBBP	4.44	4	57.1
NOTCH1	8.89	4	57.1
PTEN	12.4	12	57.1
AKT1	0	0	42.9
DNMT3A	2.67	0	42.9
EPHA3	0.889	0	42.9
ERBB2	0	0	42.9
FGFR1	2.22	0	42.9
PRDM1	0.889	0	42.9
ERBB3	1.33	8	42.9
MYC	1.33	20	42.9
KIT	1.78	24	42.9
RET	1.78	8	42.9
APC	2.22	8	42.9
PTCH1	2.22	16	42.9
TET2	3.11	12	42.9
CDK6	0.444	40	28.6
FLT4	3.56	4	42.9
ATM	5.33	12	42.9
ATRX	8	44	28.6
ASXL1	4.89	0	28.6
EP300	3.11	0	28.6
FGFR3	3.11	0	28.6
PDGFRA	3.11	28	28.6
RB1	3.56	4	28.6
TSC2	4	4	28.6
SETD2	4.89	8	28.6
HRAS	0	20	0
KDR	4	20	0
CCND2	2.67	20	0
CDK4	1.78	16	0
IKZF1	0.444	16	14.3
FBXW7	0.889	16	14.3
JAK2	0	12	14.3
FLT1	2.67	16	14.3
MCL1	1.33	12	14.3
DDR2	2.22	12	14.3
KRAS	0.444	12	0
NF1	4.89	8	14.3

Supplemental Table 2. Correlated molecular features by BRAF, age, and pathology features.

Gene	Variable	Category	Residuals	Pvalue
ATRX	Subtype	Astro, IDH-mt	7.74896	0*
CDK6	BRAFClass	Gain	8.28496	0*
EGFR	BRAFClass	Other	4.76152	0*
EZH2	BRAFClass	Gain	13.1434	0*
HRAS	BRAFClass	Gain	5.82818	0*
HRAS	Subtype	Astro, IDH-mt	5.12198	0*
IDH1	Subtype	Astro, IDH-mt	5.66663	0*
JAK2	Subtype	Astro, IDH-mt	4.76264	0*
MET	BRAFClass	Gain	5.60577	0*
MYC	BRAFClass	Gain	4.81475	0*
NF1	BRAFClass	Class II	4.69852	0*
NF1	BRAFClass	Class III	9.30047	0*
PTEN	Grade	4	6.48847	0*
PTEN	Age	>50	6.73862	0*
PTEN	Subtype	GBM, IDH-wt	6.54623	0*
SMO	BRAFClass	Gain	11.8351	0*
TP53	BRAFClass	Gain	5.43987	0*
TP53	Subtype	Astro, IDH-mt	10.1851	0*
MYC	Subtype	Astro, IDH-mt	4.44705	1e-04*
RB1	Subtype	Astro, IDH-mt	4.44705	1e-04*
SMO	Subtype	Astro, IDH-mt	4.38835	1e-04*
MET	Grade	4	4.27699	2e-04*
NF1	Age	>50	4.20281	2e-04*
PTEN	Age	<18	-4.2212	2e-04*
FGFR4	BRAFClass	Other	4.18746	3e-04*
IKZF1	BRAFClass	Gain	4.19506	3e-04*
JAK2	BRAFClass	Gain	4.19506	3e-04*
MSH2	BRAFClass	Other	4.18746	3e-04*
MSH6	Subtype	Astro, IDH-mt	4.12979	4e-04*
EP300	BRAFClass	Other	4.11355	5e-04*
GATA3	BRAFClass	Gain	4.10254	5e-04*
GNA11	BRAFClass	Gain	4.10254	5e-04*
CDK4	BRAFClass	Gain	4.03375	7e-04*
CDKN2A	BRAFClass	Class II	4.00046	8e-04*
EGFR	Age	>50	3.88911	8e-04*
ERBB2	BRAFClass	Class II	4.00046	8e-04*
SRC	BRAFClass	Class II	4.00046	8e-04*
SMO	BRAFClass	Class I	-3.9473	9e-04*
EPHA3	BRAFClass	Other	3.92309	0.001*
CCND2	Subtype	Astro, IDH-mt	3.89538	0.0012*
MET	Age	>50	3.78736	0.0012*

FLT4	Grade	3	3.75298	0.0014*
EGFR	Subtype	GBM, IDH-wt	3.81688	0.0016*
EGFR	Grade	4	3.68527	0.0018*
PDGFRA	BRAFClass	Gain	3.79005	0.0018*
TP53	Grade	1	-3.685	0.0018*
MLH1	Subtype	PXA	3.7744	0.0019*
TP53	Subtype	PA	-3.7797	0.0019*
EZH2	BRAFClass	Class I	-3.7332	0.0023*
BCL2	BRAFClass	Class III	3.71729	0.0024*
MLH1	Grade	3	3.54283	0.0032*
TP53	BRAFClass	Class I	-3.625	0.0035*
AURKB	Subtype	Astro, IDH-mt	3.59908	0.0038*
CDK6	Subtype	Astro, IDH-mt	3.60408	0.0038*
MAP2K4	Subtype	Astro, IDH-mt	3.59908	0.0038*
PIK3R1	Subtype	Astro, IDH-mt	3.59908	0.0038*
CDH1	Subtype	PXA	3.53032	0.005*
NF1	Subtype	GBM, IDH-wt	3.52247	0.0051*
PTCH1	BRAFClass	Other	3.50966	0.0054*
EZH2	Grade	4	3.35543	0.0063*
TP53	Grade	3	3.3272	0.007*
CDH1	Grade	3	3.2798	0.0083*
PIK3CA	Grade	4	3.28148	0.0083*
PTEN	Grade	2	-3.2449	0.0094*
EZH2	Grade	1	-3.2383	0.0096*
FLT4	Subtype	Astro, IDH-mt	3.34884	0.0097*
MSH6	BRAFClass	Other	3.34994	0.0097*
EZH2	BRAFClass	Fusion	-3.3352	0.0102*
EZH2	Subtype	PA	-3.3029	0.0115*
PDGFRA	Grade	4	3.15635	0.0128*
ARID2	Subtype	Astro, IDH-mt	3.25808	0.0135*
BRAF	Subtype	Astro, IDH-mt	-3.2581	0.0135*
BRIP1	Subtype	Astro, IDH-mt	3.25808	0.0135*
CTNNB1	Subtype	Astro, IDH-mt	3.25808	0.0135*
PRKAR1A	Subtype	Astro, IDH-mt	3.25808	0.0135*
SMO	Age	<18	-3.1365	0.0137*
CCND2	Subtype	HGG	3.24336	0.0142*
ATRX	Grade	3	3.11769	0.0146*
TP53	Age	<18	-3.106	0.0152*
CDKN2C	Age	>50	3.0778	0.0167*
PTEN	Grade	1	-3.066	0.0174*
AKT1	BRAFClass	Class II	3.17311	0.0181*
IDH1	BRAFClass	Class II	3.17311	0.0181*
MEN1	BRAFClass	Class II	3.17311	0.0181*

MTOR	BRAFClass	Class II	3.17311	0.0181*
RARA	BRAFClass	Class II	3.17311	0.0181*
KIT	Grade	4	3.04539	0.0186*
NF1	Grade	4	3.04539	0.0186*
MET	BRAFClass	Class I	-3.1573	0.0191*
KRAS	BRAFClass	Gain	3.15559	0.0192*
MCL1	BRAFClass	Gain	3.15349	0.0194*
PTEN	Subtype	PA	-3.1505	0.0196*
GATA3	Age	18-34	3.02002	0.0202*
PTEN	BRAFClass	Class III	3.13272	0.0208*
EZH2	Subtype	Astro, IDH-mt	3.12929	0.021*
SMO	Age	35-50	2.98316	0.0228*
MSH2	Grade	4	2.96601	0.0241*
CDK4	Age	>50	2.95214	0.0252*
RET	Age	35-50	2.93199	0.0269*
SMO	Grade	1	-2.9273	0.0274*
EZH2	Age	<18	-2.922	0.0278*
ATRX	Age	<18	-2.9187	0.0281*
SMO	BRAFClass	Fusion	-3.0343	0.0289*
TET2	Subtype	HGG	3.02393	0.0299*
SMO	Subtype	PA	-2.9986	0.0325*
NF1	BRAFClass	Class I	-2.9459	0.0386*
BCL6	BRAFClass	Other	2.94241	0.0391*
CDKN2C	BRAFClass	Other	2.94241	0.0391*
ESR1	BRAFClass	Other	2.94241	0.0391*
MAP2K4	BRAFClass	Other	2.94241	0.0391*
MDM2	BRAFClass	Other	2.94241	0.0391*
MTOR	BRAFClass	Other	2.94241	0.0391*
SUFU	BRAFClass	Other	2.94241	0.0391*
IDH1	BRAFClass	Class III	2.93428	0.0401*
MTOR	Subtype	HGG	2.93428	0.0401*
PTPN11	BRAFClass	Class III	2.93428	0.0401*
PTCH1	Grade	4	2.80494	0.0403*
EGFR	BRAFClass	Class I	-2.9207	0.0419*
EGFR	BRAFClass	Gain	2.91489	0.0427*
EZH2	Age	35-50	2.78373	0.043*
HRAS	Age	35-50	2.78222	0.0432*
AKT3	BRAFClass	Gain	2.89493	0.0455*
CCND1	BRAFClass	Gain	2.89493	0.0455*
CDC73	BRAFClass	Gain	2.89493	0.0455*
GNAQ	BRAFClass	Gain	2.89493	0.0455*
SOCS1	BRAFClass	Gain	2.89493	0.0455*
