# Supplementary material for: Integrated molecular and clinical analysis of BRAF-mutant glioma in adults
Source: NPJ Precis Oncol. 2023 Feb 28;7:23. doi: 10.1038/s41698-023-00359-y (PMC9975216; doi:10.1038/s41698-023-00359-y)
Supplement: Supplementary file 2 — REPORTING SUMMARY [file 41698_2023_359_MOESM2_ESM.pdf]

nature portfolio

Corresponding author(s): KC Schreck and WL Bi

Last updated by author(s): Nov 29, 2022

Reporting Summary

Nature Portfolio wishes to improve the reproducibility of the work that we publish. This form provides structure for consistency and transparency in reporting. For further information on Nature Portfolio policies, see our Editorial Policies and the Editorial Policy Checklist.

Statistics

For all statistical analyses, confirm that the following items are present in the figure legend, table legend, main text, or Methods section.

n/a	Confirmed	
☐	☒	The exact sample size (*n*) for each experimental group/condition, given as a discrete number and unit of measurement
☒	☐	A statement on whether measurements were taken from distinct samples or whether the same sample was measured repeatedly
☐	☒	The statistical test(s) used AND whether they are one- or two-sided<br>*Only common tests should be described solely by name; describe more complex techniques in the Methods section.*
☐	☒	A description of all covariates tested
☐	☒	A description of any assumptions or corrections, such as tests of normality and adjustment for multiple comparisons
☐	☒	A full description of the statistical parameters including central tendency (e.g. means) or other basic estimates (e.g. regression coefficient) AND variation (e.g. standard deviation) or associated estimates of uncertainty (e.g. confidence intervals)
☒	☐	For null hypothesis testing, the test statistic (e.g. *F*, *t*, *r*) with confidence intervals, effect sizes, degrees of freedom and *P* value noted<br>*Give P values as exact values whenever suitable.*
☒	☐	For Bayesian analysis, information on the choice of priors and Markov chain Monte Carlo settings
☒	☐	For hierarchical and complex designs, identification of the appropriate level for tests and full reporting of outcomes
☐	☒	Estimates of effect sizes (e.g. Cohen's *d*, Pearson's *r*), indicating how they were calculated

Our web collection on statistics for biologists contains articles on many of the points above.

Software and code

Policy information about availability of computer code

Data collection	Patients aged >18 years at the time of diagnosis with a glioma containing a BRAF alteration on sequencing were identified at the Dana-Farber/ Harvard Cancer Center (DFCI), Johns Hopkins Hospital (JHH), and 2 public repositories: The Cancer Genome Atlas Program (TCGA) and Project Genomics Evidence Neoplasia Information Exchange (GENIE).

Data analysis	All statistical analyses were performed using R statistical software, version 3.6.3 (R Foundation for Statistical Computing; www.r-project.org). Clustering analysis was performed using 136 genes with cross-coverage across all non-hypermutated samples with genomic data. For each sample, genes were binarized as either altered or non-altered. Logistic principal component analysis (PCA) was used implemented using the logisticPCA package (CRAN) to reduce the dimensionality of the binary alterations matrix (genes x samples) and k-means clustering was performed on the first 25 logistic principal components. We used the silhouette and elbow methods to determine the optimal number of k-means clusters.<br><br>Correlations between BRAF alteration types, patient characteristics, and other genomic alterations (binarized as present or absent for each gene) were evaluated using Chi-squared test constructed from pairwise contingency tables. Strength of association was assessed using Cramer's V statistic. Post-hoc analysis was applied on significant associations to dissect directions of effect.<br><br>Associations between clinical, histopathologic, and molecular covariates of interest and overall survival were assessed by univariate analysis using Cox proportional hazards models. Correction for multiple comparisons was performed using the Benjamini–Hochberg method. Variables with an adjusted false discovery rate (FDR) <0.05 and altered in at least five individual patients were reported as significant. Overall survival rates were compared by the Kaplan-Meier method with censoring to date of last follow-up and significance determined by log-rank test. All survival analyses were performed using the survival (CRAN) and survminer (CRAN) packages.

For manuscripts utilizing custom algorithms or software that are central to the research but not yet described in published literature, software must be made available to editors and reviewers. We strongly encourage code deposition in a community repository (e.g. GitHub). See the Nature Portfolio guidelines for submitting code & software for further information.

Data

Policy information about availability of data

All manuscripts must include a data availability statement. This statement should provide the following information, where applicable:

- Accession codes, unique identifiers, or web links for publicly available datasets
- A description of any restrictions on data availability
- For clinical datasets or third party data, please ensure that the statement adheres to our policy

Datasets from TCGA used in this project are publicly available using the National Cancer Institute Genomic Data Commons (GDC) Data Portal (https://portal.gdc.cancer.gov/). TCGA data are listed under the Project IDs "TCGA-LGG" and "TCGA-GBM." Datasets from GENIE used in this project are publicly available using the cBioPortal (https://genie.cbioportal.org/) from the "GENIE Cohort v12.0-public" study. Consent for public data sharing was not obtained from DFCI and JHH patients, so datasets from DFCI and Johns Hopkins Hospital are available upon data usage agreement from the corresponding authors.

Human research participants

Policy information about studies involving human research participants and Sex and Gender in Research.

Reporting on sex and gender	*Use the terms sex (biological attribute) and gender (shaped by social and cultural circumstances) carefully in order to avoid confusing both terms. Indicate if findings apply to only one sex or gender; describe whether sex and gender were considered in study design whether sex and/or gender was determined based on self-reporting or assigned and methods used. Provide in the source data disaggregated sex and gender data where this information has been collected, and consent has been obtained for sharing of individual-level data; provide overall numbers in this Reporting Summary. Please state if this information has not been collected. Report sex- and gender-based analyses where performed, justify reasons for lack of sex- and gender-based analysis.*
Population characteristics	*Describe the covariate-relevant population characteristics of the human research participants (e.g. age, genotypic information, past and current diagnosis and treatment categories). If you filled out the behavioural & social sciences study design questions and have nothing to add here, write "See above."*
Recruitment	*Describe how participants were recruited. Outline any potential self-selection bias or other biases that may be present and how these are likely to impact results.*
Ethics oversight	*Identify the organization(s) that approved the study protocol.*

Note that full information on the approval of the study protocol must also be provided in the manuscript.

Field-specific reporting

Please select the one below that is the best fit for your research. If you are not sure, read the appropriate sections before making your selection.

☒ Life sciences ☐ Behavioural & social sciences ☐ Ecological, evolutionary & environmental sciences

For a reference copy of the document with all sections, see nature.com/documents/nr-reporting-summary-flat.pdf

Life sciences study design

All studies must disclose on these points even when the disclosure is negative.

Sample size	descriptive cohort study incorporating all eligible patients
Data exclusions	no data were excluded
Replication	n/a
Randomization	n/a
Blinding	n/a

Reporting for specific materials, systems and methods

We require information from authors about some types of materials, experimental systems and methods used in many studies. Here, indicate whether each material, system or method listed is relevant to your study. If you are not sure if a list item applies to your research, read the appropriate section before selecting a response.

Materials & experimental systems

n/a	Involved in the study	
☒	☐	Antibodies
☒	☐	Eukaryotic cell lines
☒	☐	Palaeontology and archaeology
☒	☐	Animals and other organisms
☒	☐	Clinical data
☒	☐	Dual use research of concern

Methods

n/a	Involved in the study	
☒	☐	ChIP-seq
☒	☐	Flow cytometry
☒	☐	MRI-based neuroimaging
